# Supplementary material for: GSK-3 Inhibitor Elraglusib Enhances Tumor-Infiltrating Immune Cell Activation in Tumor Biopsies and Synergizes with Anti-PD-L1 in a Murine Model of Colorectal Cancer
Source: Int J Mol Sci. 2023 Jun 29;24(13):10870. doi: 10.3390/ijms241310870 (PMC10342141; doi:10.3390/ijms241310870)
Supplement: Supplementary file 1 [file ijms-24-10870-s001.zip › ijms-2446136-supplementary.pdf]

HCT-116 Elraglusib vs. NT

Gene Symbol	Fold Change	P-val	Description	Function
BTG2	4.35	7.46E-10	BTG family, member 2	Anti-proliferative
MDM2	4.17	3.65E-11	MDM2 proto-oncogene, E3 ubiquitin protein ligase	Contributes to TP53 reguation
TP53INP1	3.62	4.21E-11	tumor protein p53 inducible nuclear protein 1	Antiproliferative / proapoptotic
LYZ	3.41	2.30E-03	lysozyme	Anti-proliferative
DRAM1	3.1	3.05E-08	DNA-damage regulated autophagy modulator 1	Proapoptotic
GADD45A	3.04	1.34E-09	growth arrest and DNA-damage-inducible, alpha	Anti-proliferative
CDKN1A	2.95	1.12E-09	cyclin-dependent kinase inhibitor 1A (p21, Cip1)	Anti-proliferative
PMAIP1	2.8	3.21E-05	phorbol-12-myristate-13-acetate-induced protein 1	Promotes activation of caspases and apoptosis
ATF3	2.73	1.04E-10	activating transcription factor 3	Anti-proliferative
FAS	2.56	2.17E-10	Fas cell surface death receptor	Proapoptotic
BLOC1S2	2.5	2.50E-08	biogenesis of lysosomal organelles complex-1, subunit 2	Proapoptotic
SESN1	2.32	3.65E-09	sestrin 1	Anti-proliferative
TNFRSF10D	2.25	1.68E-07	tumor necrosis factor receptor superfamily, member 10d	Decoy TRAIL receptor
TNFRSF10B	2.12	1.26E-08	tumor necrosis factor receptor superfamily, member 10b	Proapoptotic
KLLN	2.08	4.00E-04	killin, p53-regulated DNA replication inhibitor	Proapoptotic
AEN	2.04	3.09E-06	apoptosis enhancing nuclease	Proapoptotic
PLK3	2	2.56E-07	polo-like kinase 3	Proapoptotic
MXD1	1.94	3.00E-04	MAX dimerization protein 1	Proapoptotic
GADD45B	1.88	6.64E-06	growth arrest and DNA-damage-inducible, beta	Proapoptotic
TRIM31	1.79	3.10E-03	tripartite motif containing 31	Proapoptotic
PPP1R1C	1.78	7.50E-05	protein phosphatase 1, regulatory (inhibitor) subunit 1C	May increase cell susceptibility to TNF-induced apoptosis
NFKBIA	1.77	5.00E-04	nuclear factor of kappa light chain gene enhancer in B-cells inhibitor, alpha	Regulator of NF-kappa-B
TP53I3	1.77	3.31E-07	tumor protein p53 inducible protein 3	Proapoptotic
CSNK1G1	1.72	3.31E-06	casein kinase 1, gamma 1	Antiproliferative and proapoptotic
SUSD6	1.71	2.15E-05	sushi domain containing 6	Antiproliferative and proapoptotic
TNFRSF10A	1.68	3.00E-04	tumor necrosis factor receptor superfamily, member 10a	Proapoptotic / Regulator of NF-kappa-B
TNFAIP3	1.64	2.64E-06	tumor necrosis factor, alpha-induced protein 3	Negative regulator of NF-kappa-B
TNFRSF9	1.52	1.00E-04	tumor necrosis factor receptor superfamily, member 9	T cell costimulatory receptor 4-1BB
TNFRSF10C	1.52	3.03E-05	tumor necrosis factor receptor superfamily, member 10c	Decoy TRAIL receptor
BAK1	1.51	2.60E-03	BCL2-antagonist/killer 1	Proapoptotic
CDC25C	-1.5	1.00E-03	cell division cycle 25C	Promotes cell cycle progression
PRC1	-1.52	2.00E-04	protein regulator of cytokinesis 1	Promotes cell cycle progression
ANLN	-1.54	1.18E-05	anillin actin binding protein	Promotes cell cycle progression
BARD1	-1.56	5.00E-04	BRCA1 associated RING domain 1	Promotes cell cycle progression
IRAK1BP1	-1.57	3.16E-02	interleukin 1 receptor associated kinase 1 binding protein 1	Promotes NF-kappa-B activation
PDK1	-1.58	3.72E-05	pyruvate dehydrogenase kinase, isozyme 1	Promotes cell cycle progression
DHX32	-1.59	3.06E-06	DEAH (Asp-Glu-Ala-His) box polypeptide 32	Promotes cell cycle progression
CCNF	-1.59	2.30E-03	cyclin F	Promotes cell cycle progression
FZD3	-1.59	2.00E-04	frizzled class receptor 3	Component of the Wnt signaling pathway
ENO2	-1.6	2.55E-05	enolase 2 (gamma, neuronal)	Promotes EMT
MST1R	-1.6	1.00E-03	macrophage stimulating 1 receptor	Promotes EMT
BRCA1	-1.62	2.00E-04	breast cancer 1, early onset	Promotes cell cycle progression
FASN	-1.64	2.00E-04	fatty acid synthase	Promotes cell proliferation
ARHGEF39	-1.67	2.00E-04	Rho guanine nucleotide exchange factor 39	Promotes cell proliferation
PRR11	-1.67	2.00E-04	proline rich 11	Promotes cell cycle progression
SOX4	-1.72	1.18E-05	SRY box 4	Induces tumor cell resistance to cytotoxic T cells
TTK	-1.72	7.34E-06	TTK protein kinase	Promotes cell cycle progression
CMTM4	-1.84	1.20E-06	CKLF-like MARVEL transmembrane domain containing 4	Protects PD-L1 from being polyubiquitinated and targeted for degradation
FANCD2	-1.86	1.27E-07	Fanconi anemia complementation group D2	Promotes cell cycle progression
FOXC1	-1.88	1.89E-08	forkhead box C1	Promotes cell proliferation / promotes EMT
CDCA3	-1.91	1.46E-05	cell division cycle associated 3	Promotes cell proliferation
AURKB	-1.93	3.44E-06	aurora kinase B	Promotes cell cycle progression
UHRF1	-1.97	4.00E-04	ubiquitin-like with PHD and ring finger domains 1	Promotes cell cycle progression
MKI67	-2.02	2.40E-03	marker of proliferation Ki-67	Promotes cell proliferation
NEK2	-2.21	5.50E-09	NIMA-related kinase 2	Inhibition sensitizes PD-L1 blockade / promotes cell proliferation

Supplementary Table S1. HCT-116 CRC cell microarray analysis. HCT-116 CRC cells were treated with 1 μM elraglusib for 24 hours and treated versus untreated control samples were compared in triplicate via microarray analysis (*N*=3). Table showing differentially expressed genes of interest with their corresponding fold changes, *p* values, descriptions, and functions. Genes highlighted in yellow are known p53 targets. Genes are ordered by fold change and were calculated using a fold change cutoff of >1.5, <–1.5, and a minimum *p* value of <0.05.

HT-29 Elraglusib vs. NT

Gene Symbol	Fold Change	P-val	Description	Function
EGR1	5.41	3.81E-12	early growth response 1	Activates tumor supressor p53
B2M	2.41	6.88E-07	beta-2-microglobulin	May shape immune landscape
AEN	2.33	1.76E-07	apoptosis enhancing nuclease	Proapoptotic
TNFRSF12A	1.9	9.60E-03	tumor necrosis factor receptor superfamily, member 12A	Proapoptotic
TRAIP	1.83	4.43E-05	TRAF interacting protein	Regulator of NF-kappa-B
NCR3LG1	1.78	2.00E-03	natural killer cell cytotoxicity receptor 3 ligand 1	Triggers NCR3-dependent NK cell activation and cytotoxicity
SOCS7	1.74	1.74E-06	suppressor of cytokine signaling 7	Anti-proliferative
CDKN1A	1.72	2.00E-04	cyclin-dependent kinase inhibitor 1A (p21, Cip1)	Anti-proliferative
SMAD3	1.69	7.01E-06	SMAD family member 3	Anti-proliferative
MDM4	1.68	8.02E-07	MDM4, p53 regulator	Contributes to TP53 reguation
BCCIP	1.61	1.24E-05	BRCA2 and CDKN1A interacting protein	Anti-proliferative
CCAR1	1.56	1.40E-03	cell division cycle and apoptosis regulator 1; small nucleolar RNA, C/D box 98	Proapoptotic
SFN	1.54	1.06E-02	stratifin	Anti-proliferative / proapoptotic
CRLF3	1.5	1.20E-03	cytokine receptor-like factor 3	Anti-proliferative
TNIK	-1.51	9.00E-04	TRAF2 and NCK interacting kinase	Promotes cell proliferation
BRAF	-1.53	2.70E-03	B-Raf proto-oncogene, serine/threonine kinase	Promotes cell proliferation
CD276	-1.56	1.00E-04	CD276 molecule	Suppresses antitumor activity
BTN3A2	-1.58	4.70E-05	butyrophilin, subfamily 3, member A2	Inhibits the release of IFNG from activated T-cells
EAPP	-1.58	8.36E-07	E2F-associated phosphoprotein	Promotes cell proliferation
JAK1	-1.58	2.94E-05	Janus kinase 1	Promotes cell proliferation
PDS5B	-1.58	5.10E-03	PDS5 cohesin associated factor B	Promotes cell proliferation
MCIDAS	-1.61	1.46E-05	multiciliate differentiation and DNA synthesis associated cell cycle protein	Promotes cell cycle progression
FADD	-1.66	5.79E-05	Fas (TNFRSF6)-associated via death domain	Regulator of NF-kappa-B
HIP1	-1.67	6.00E-04	huntingtin interacting protein 1	Antiapoptotic
IL17RA	-1.68	1.02E-05	interleukin 17 receptor A	Regulator of NF-kappa-B
MYD88	-1.68	2.00E-04	myeloid differentiation primary response 88	Regulator of NF-kappa-B
CDCA3	-1.7	3.00E-04	cell division cycle associated 3	Promotes cell proliferation
DYNC1H1	-1.7	9.20E-05	dynein, cytoplasmic 1, heavy chain 1	Promotes cell cycle progression
ERBB2IP	-1.7	1.81E-02	erbb2 interacting protein	Regulator of NF-kappa-B
FZD7	-1.71	8.02E-05	frizzled class receptor 7	Component of the Wnt signaling pathway
CDC45	-1.75	6.40E-06	cell division cycle 45	Promotes cell cycle progression
PIM1	-1.75	2.36E-05	Pim-1 proto-oncogene, serine/threonine kinase	Antiapoptotic
SGK1	-1.75	5.24E-08	serum/glucocorticoid regulated kinase 1	Antiapoptotic
UHRF1	-1.75	5.00E-04	ubiquitin-like with PHD and ring finger domains 1	Promotes cell cycle progression
MTA3	-1.84	4.07E-09	metastasis associated 1 family member 3	Promotes EMT
ITGB6	-1.91	3.26E-07	integrin beta 6	Regulator of TGF-β Signaling
IL17RB	-2.03	6.33E-06	interleukin 17 receptor B	Regulator of NF-kappa-B
AGGF1	-2.21	2.00E-04	angiogenic factor with G-patch and FHA domains 1	Promotes EMT
CDK2	-2.23	6.11E-09	cyclin-dependent kinase 2	Promotes cell cycle progression
TNFSF15	-2.32	2.58E-07	tumor necrosis factor (ligand) superfamily, member 15	Regulator of NF-kappa-B
CDC25C	-2.45	3.43E-08	cell division cycle 25C	Promotes cell cycle progression
CCNE1	-2.6	1.13E-08	cyclin E1	Promotes cell cycle progression
CD14	-2.63	2.82E-07	CD14 molecule	Regulator of NF-kappa-B
NFKBIZ	-2.68	6.65E-10	nuclear factor of kappa light polypeptide gene enhancer in B-cells inhibitor, zeta	Regulator of NF-kappa-B
CDK1	-3.05	5.79E-07	cyclin-dependent kinase 1	Promotes cell cycle progression
E2F8	-3.1	7.74E-07	E2F transcription factor 8	Promotes EMT
BCL6	-3.43	8.65E-11	B-cell CLL/lymphoma 6	Antiapoptotic
E2F7	-3.82	1.32E-07	E2F transcription factor 7	Promotes EMT / antiapoptotic
TGFBR3	-4.82	1.01E-11	transforming growth factor beta receptor III	Regulates TGF-β Signaling
BARD1	-5.06	2.65E-10	BRCA1 associated RING domain 1	Promotes cell cycle progression
NFKBIA	-5.25	3.00E-10	nuclear factor of kappa light polypeptide gene enhancer in B-cells inhibitor, alpha	Regulator of NF-kappa-B
CCNE2	-5.83	6.53E-10	cyclin E2	Promotes cell cycle progression
MAP3K1	-6.1	2.49E-13	mitogen-activated protein kinase kinase kinase 1, E3 ubiquitin protein ligase	Regulator of NF-kappa-B
TRIB1	-9.14	8.08E-12	tribbles pseudokinase 1	Antiapoptotic

Supplementary Table S2. HT-29 CRC cell microarray analysis. HT-29 CRC cells were treated with 1 μM elraglusib for 24 hours and treated versus untreated control samples were compared in triplicate via microarray analysis (N=3). Table showing differentially expressed genes of interest with their corresponding fold changes, p values, descriptions, and functions. Genes highlighted in yellow are known p53 targets. Genes are ordered by fold change and were calculated using a fold change cutoff of >1.5, <−1.5, and a minimum p value of <0.05.

KM12C Elraglusib vs. NT

Gene Symbol	Fold Change	P-val	Description	Function
IL32	1.55	4.00E-03	interleukin 32	Regulator of NF-kappa-B
GZMA	1.53	2.16E-02	granzyme A	Triggers pyroptosis
TNFRSF12A	1.53	3.13E-02	tumor necrosis factor receptor superfamily, member 12A	Proapoptotic
TRAIP	1.53	9.80E-03	TRAF interacting protein	Regulator of NF-kappa-B
BIK	1.51	1.30E-03	BCL2-interacting killer (apoptosis-inducing)	Proapoptotic
CDCA2	-1.51	1.70E-03	cell division cycle associated 2	Promotes cell cycle progression
IGF2BP2	-1.51	5.00E-04	insulin-like growth factor 2 mRNA binding protein 2	Promotes cell cycle progression / antiapoptotic
TRAF5	-1.52	5.60E-03	TNF receptor-associated factor 5	Regulator of NF-kappa-B
CXCL1	-1.53	2.00E-04	chemokine (C-X-C motif) ligand 1	Promotes EMT
MKI67	-1.53	7.50E-03	marker of proliferation Ki-67	Promotes cell proliferation
TNFRSF10A	-1.54	6.50E-03	tumor necrosis factor receptor superfamily, member 10a	Proapoptotic / Regulator of NF-kappa-B
BCL9	-1.55	3.49E-02	B-cell CLL/lymphoma 9	Promotes the Wnt signaling pathway
CDC25C	-1.55	5.00E-04	cell division cycle 25C	Promotes cell cycle progression
TNFRSF1B	-1.56	2.65E-02	tumor necrosis factor receptor superfamily, member 1B	Antiapoptotic
CCNE1	-1.57	5.79E-05	cyclin E1	Promotes cell cycle progression
TRAF6	-1.57	1.00E-04	TNF receptor-associated factor 6, E3 ubiquitin protein ligase	Regulator of NF-kappa-B
BRD3	-1.58	1.00E-04	bromodomain containing 3	Antiapoptotic
CCND2	-1.58	7.90E-03	cyclin D2	Promotes cell cycle progression
MTBP	-1.58	2.92E-02	MDM2 binding protein	Contributes to TP53 regaution
TNIK	-1.59	4.00E-04	TRAF2 and NCK interacting kinase	Promotes the Wnt signaling pathway
AGGF1	-1.6	1.23E-02	angiogenic factor with G-patch and FHA domains 1	Promotes EMT
IRF2BP2	-1.6	3.00E-04	interferon regulatory factor 2 binding protein 2	Promotes EMT
MDM2	-1.61	1.20E-03	MDM2 proto-oncogene, E3 ubiquitin protein ligase	Contributes to TP53 regaution
TAB3	-1.63	5.00E-04	TGF-beta activated kinase 1/MAP3K7 binding protein 3	Regulator of NF-kappa-B
TNFRSF11A	-1.64	1.23E-05	tumor necrosis factor receptor superfamily, member 11a, NFKB activator	Regulator of NF-kappa-B
BRCA1	-1.71	8.00E-04	breast cancer 1, early onset	Promotes cell cycle progression
IL27RA	-1.72	7.00E-04	interleukin 27 receptor, alpha	Binds immunomodulatory cytokine IL-27
MTDH	-1.74	8.48E-05	metadherin	Regulator of NF-kappa-B
FZD3	-1.76	8.90E-05	frizzled class receptor 3	Component of the Wnt signaling pathway
MET	-1.79	1.77E-05	MET proto-oncogene, receptor tyrosine kinase	Promotes EMT
ERBB2IP	-1.88	9.00E-04	erbb2 interacting protein	Regulator of NF-kappa-B
IL6ST	-1.88	8.13E-05	interleukin 6 signal transducer	Component of the IL-6 signaling pathway
NRP1	-1.88	1.70E-05	neuropilin 1	Promotes EMT
MAP3K1	-1.92	1.31E-05	mitogen-activated protein kinase kinase kinase 1, E3 ubiquitin protein ligase	Regulator of NF-kappa-B
CDK1	-2	1.00E-04	cyclin-dependent kinase 1	Promotes cell cycle progression
JMY	-2.03	1.15E-05	junction mediating and regulatory protein, p53 cofactor	Contributes to TP53 regaution
BRAF	-2.05	2.00E-04	B-Raf proto-oncogene, serine/threonine kinase	Promotes cell proliferation
CD109	-2.09	3.00E-04	CD109 molecule	Regulates TGF-β Signaling
TGFBR2	-2.11	7.17E-07	transforming growth factor beta receptor II	Regulates TGF-β Signaling
LTBP1	-2.24	4.70E-06	latent transforming growth factor beta binding protein 1	Regulates TGF-β Signaling
TLR3	-2.25	6.24E-06	toll-like receptor 3	Regulator of NF-kappa-B
GDF15	-2.29	6.00E-04	growth differentiation factor 15	Promotes EMT
TRIB1	-2.37	8.89E-06	tribbles pseudokinase 1	Antiapoptotic
E2F7	-2.5	9.13E-05	E2F transcription factor 7	Promotes EMT / antiapoptotic
NFKBIA	-2.51	3.25E-07	nuclear factor of kappa light polypeptide gene enhancer in B-cells inhibitor, alpha	Regulator of NF-kappa-B
CCNE2	-2.64	5.46E-07	cyclin E2	Promotes cell cycle progression
BARD1	-3.03	1.59E-07	BRCA1 associated RING domain 1	Promotes cell cycle progression
E2F8	-3.16	3.26E-07	E2F transcription factor 8	Promotes EMT
MAP2K6	-3.66	1.13E-09	mitogen-activated protein kinase kinase 6	Antiapoptotic
TGFBR3	-3.72	7.53E-10	transforming growth factor beta receptor III	Regulates TGF-β Signaling

Supplementary Table S3. KM12C CRC cell microarray analysis. KM12C CRC cells were treated with 1 μM elraglusib for 24 hours and treated versus untreated control samples were compared in triplicate via microarray analysis (*N*=3). Table showing differentially expressed genes of interest with their corresponding fold changes, *p* values, descriptions, and functions. Genes highlighted in yellow are known p53 targets. Genes are ordered by fold change and were calculated using a fold change cutoff of >1.5, <−1.5, and a minimum *p* value of <0.05.

NK-92 Elraglusib vs. NT

Gene Symbol	Fold Change	P-val	Description	Function
RNY5	3.32	0.027	RNA, Ro-associated Y5	May modulate NF-κB activity
RAB38	1.65	5.44E-05	RAB38, member RAS oncogene family	Promotes cellular proliferation
TNFSF14	1.5	0.043	tumor necrosis factor (ligand) superfamily, member 14	Stimulates T cell proliferation
WNK1	1.5	0.0119	WNK lysine deficient protein kinase 1	Controls immune cell adhesion and migration
NAP1L1	-1.51	0.0278	nucleosome assembly protein 1-like 1	May modulate NF-κB activity
MIR186	-1.52	0.0132	microRNA 186	Proapoptotic
ITGB8	-1.53	0.0059	integrin beta 8	Activates latent TGFβ to supress immune responses
S100A12	-1.78	0.0407	S100 calcium binding protein A12	Proapototic

Supplementary Table S4. NK-92 immune cell microarray analysis. NK-92 immune cells were treated with 1 μM elraglusib for 24 hours and treated versus untreated control samples were compared in triplicate via microarray analysis (*N*=3). Table showing differentially expressed genes of interest with their corresponding fold changes, *p* values, descriptions, and functions. Genes are ordered by fold change and were calculated using a fold change cutoff of >1.5, <−1.5, and a minimum *p* value of <0.05.

TALL-104 Elraglusib vs. NT

Gene Symbol	Fold Change	P-val	Description	Function
RNY4	2.19	0.0218	RNA, Ro-associated Y4	May modulate NF-κB activity
RNY5	1.66	0.0416	RNA, Ro-associated Y5	May modulate NF-κB activity
BCL2A1	1.63	0.023	BCL2-related protein A1	Antiapoptotic
CKS1B	1.6	0.013	CDC28 protein kinase regulatory subunit 1B	Promotes cell proliferation
STX19	1.58	0.0004	syntaxin 19	Involved in cytotoxic granule exocytosis
VAMP8	1.56	0.0193	vesicle associated membrane protein 8	Involved in cytotoxic granule exocytosis
KIF7	1.56	0.0091	kinesin family member 7	Required for T cell development and MHC expression
CCL3	1.52	0.022	chemokine (C-C motif) ligand 3	Recruits and enhances proliferation of CD8+ T cells
ORAI3	1.51	0.0387	ORAI calcium release-activated calcium modulator 3	Promotes cell proliferation
CD84	-1.53	0.0318	CD84 molecule	Regulator of immune cell function
PPARA	-1.54	0.0074	peroxisome proliferator-activated receptor alpha	Regulator of immune cell function
PTPN3	-1.56	0.0239	protein tyrosine phosphatase, non-receptor type 3	Inhibitory immune checkpoint
ACVR1B	-1.61	0.0205	activin A receptor type IB	Regulates TGFβ signaling
PTPN14	-1.62	0.0041	protein tyrosine phosphatase, non-receptor type 14	Regulates TGFβ signaling
HSPA1A	-1.63	0.0391	heat shock 70kDa protein 1B; heat shock 70kDa protein 1A	Proapoptotic
DUSP6	-1.66	0.0013	dual specificity phosphatase 6	Regulator of immune cell function
UBE3A	-1.73	0.0286	ubiquitin protein ligase E3A	Proapoptotic
CCR8	-2.14	0.0004	chemokine (C-C motif) receptor 8	Marker of regulatory T cells
CAMK1D	-2.5	0.0256	calcium/calmodulin-dependent protein kinase ID	Key modulator of tumor-intrinsic immune resistance

Supplementary Table S5. TALL-104 immune cell microarray analysis. TALL-104 immune cells were treated with 1 μM elraglusib for 24 hours and treated versus untreated control samples were compared in triplicate via microarray analysis (*N*=3). Table showing differentially expressed genes of interest with their corresponding fold changes, *p* values, descriptions, and functions. Genes are ordered by fold change and were calculated using a fold change cutoff of >1.5, <−1.5, and a minimum *p* value of <0.05.

Test code	Isotype control	Elraglusib	αPD-1	αPD-L1	αPD-1 + Elraglusib	αPD-L1 + Elraglusib
BUN (mg/dL)	23	27	28	23	23	23
CREA (mg/dL)	0.2	0.2	0.2	<0.2	0.2	0.2
GLU (mg/dL)	217	230	340	176	240	292
NA (mmol/L)	148	146	146	149	147	147
K (mmol/L)	6.8	>10.0	6.1	6.9	6.2	5.7
CL (mmol/L)	110	111	112	116	111	114
ALP (U/L)	38	4	46	21	36	83
ALT (U/L)	16	26	23	42	26	36
AST (U/L)	266	198	197	883	217	218
TBIL (mg/dL)	0.2	0.5	0.1	0.4	0.2	0.1
DBIL (mg/dL)	0	0	0	0.1	0	0
LDH (U/L)	867	2230	710	>12000	771	447
CPK (U/L)	476	1968	1396	447	653	1477
GGT (U/L)	0	0	0	0	0	0
TPRO (g/dL)	4.2	4.5	4.2	3.7	4.2	4.3
ALB (g/dL)	2.4	2.7	2.4	2	2.5	2.6
CA (mg/dL)	10	0.4	9.6	7.3	10.2	8.6
PHOS (mg/dL)	8.4	8.2	11.4	6.2	7.5	8.9
MG (mg/dL)	2.5	1.2	2.9	2.1	2.5	2.5
CHOL (mg/dL)	90	101	81	81	83	67
TRIG (mg/dL)	269	136	159	434	169	75
AMY (U/L)	265	275	307	241	318	442
LIP (U/L)	49	59	55	52	42	66
WBC (10^3/μL)	--	--	2.96	1.8	3.75	2.68
RBC (10^6/μL)	--	--	8.86	8.16	8.5	8.85
HB (g/dL)	--	--	13	11.9	12.7	13.2
HCT (%)	--	--	50.6	46.9	49	51
MCV (fL)	--	--	57.1	57.5	57.6	57.6
MCH (pg)	--	--	14.6	14.6	14.9	14.9
MCHC (g/dL)	--	--	25.6	25.3	25.9	25.8
PLT (10^3/μL)	--	--	403	691	401	471
NEU% (%)	--	--	55.8	29.4	60.1	50.8
NEU (10^3/μL)	--	--	1.65	0.53	2.26	1.36
LYM% (%)	--	--	34.7	57.3	33.1	39.6
LYM (10^3/μL)	--	--	1.03	1.03	1.24	1.06
MON% (%)	--	--	3	3.3	1.2	2.2
MON (10^3/μL)	--	--	0.09	0.06	0.04	0.06
EOS% (%)	--	--	3	5.1	2.8	4.3
EOS (10^3/μL)	--	--	0.09	0.09	0.11	0.11
BAS% (%)	--	--	0.8	0.5	0.3	1.4
BAS (10^3/μL)	--	--	0.02	0.01	0.01	0.04
LUC% (%)	--	--	2.6	4.4	2.5	3.2
LUC (10^3/μL)	--	--	0.08	0.08	0.1	0.09

Supplementary Table S6. Murine serum chemistry analysis does not show treatment-related toxicity. Whole blood from long-term mice sacrificed was submitted for serum chemistry analysis. Results are shown from the complete metabolic panel and complete blood count with differential. ALB: Albumin, ALP: Alkaline Phosphatase, ALT: Alanine aminotransferase, AMY: Amylase, ANIS: Anisocytosis, AST:

Aspartate aminotransferase, ATYP: Atypical Lymphs, BAS: Absolute Basophils, BAS%: % Basophils, BUN: Urea Nitrogen, CA: Calcium, CHOL: Cholesterol, CL: Chloride, CPK: Creatine kinase, CPLT: Clumped Platelets, CREA: Creatinine, DBIL: Direct Bilirubin, EOS: Absolute Eosinophils, EOS% : % Eosinophils, GGT: Gamma-glutamyl Transferase, GLU: Glucose, HB: Hemoglobin, HCT: Hematocrit, HJB: Howell-Jolly Bodies, HYPO: Hypochromasia, HYPR: Hyperchromasia, K: Potassium, LDH: Lactate Dehydrogenase, LIP: Lipase, LPLT: Large Platelets, LUC: Absolute Large Unstained Cells, LUC%: % Large Unstained Cells, LYM: Absolute Lymphocytes, LYM%: % Lymphocytes, MAC: Macrocytosis, MCH: Mean Corpuscular Hemoglobin, MCHC: Mean Corpuscular Hemoglobin Count, MCV: Mean Corpuscular Volume, MG: Magnesium, MIC: Microcytosis, MON: Absolute Monocytes, MON%: % Monocytes, NA: Sodium, NEU: Absolute Neutrophils, NEU%: % Neutrophils, PHOS: Inorganic Phosphorus, PLT: Platelet Count, POLK: Poikilocytosis, RBC: Red Blood Cell Count, TBIL: Total Bilirubin, TPRO: Total Protein, TRIG: Triglyceride, WBC: White Blood Cell Count.

Subject ID	Tumor type	Elraglusib dose (mg) cycle 1, cycle 2	Elraglusib dose (mg/kg)	Number of cycles	Age at enrollment (years)	Sex	Race	Ethnicity	PFS (days)	OS (days)
05001	Pancreas	67	1	1	61	F	White/Caucasian	Not Hispanic/Latino	UN	UN
05002	Appendix	47	1	1	59	F	Other	Hispanic or Latino	UN	UN
05003	Colorectal	58	1	1	56	F	White/Caucasian	Not Hispanic/Latino	26	26
05004	Colorectal	76	1	1	60	M	White/Caucasian	Not Hispanic/Latino	28	UN
05005	Colorectal	71, 130	1	2	68	M	White/Caucasian	Not Hispanic/Latino	99	105
05006	HCC	205	2	1	59	M	White/Caucasian	Not Hispanic/Latino	234	234
05009	Cholangiocarcinoma	144	2	1	60	M	White/Caucasian	Not Hispanic/Latino	46	UN
05010	Colorectal	182	2	1	51	M	White/Caucasian	Not Hispanic/Latino	UN	UN
05011	NSCLC	105	2	1	51	F	White/Caucasian	Not Hispanic/Latino	31	39
05013	Colorectal	235	3.3	1	70	F	White/Caucasian	Not Hispanic/Latino	130	UN
05016	Colorectal	219	3.3	1	33	F	White/Caucasian	Not Hispanic/Latino	UN	UN
05017	Colorectal	603	5	1	61	F	Black or African American	Not Hispanic/Latino	83	UN
05018	NSCLC	305	5	1	73	F	White/Caucasian	Not Hispanic/Latino	41	UN
05019	Appendix	450	7	1	63	F	White/Caucasian	Not Hispanic/Latino	UN	UN
05020	Desmoid	741	7	1	28	F	White/Caucasian	Not Hispanic/Latino	UN	UN
05024	Appendix	354	7	1	71	F	White/Caucasian	Not Hispanic/Latino	41	UN
05038	Pancreas	766	9.3	1	70	F	White/Caucasian	Not Hispanic/Latino	UN	UN
05051	ATLL	673	12.37	1	45	M	Black or African American	Not Hispanic/Latino	UN	UN
05055	Leiomyosarcoma	518	12.37	1	67	M	White/Caucasian	Not Hispanic/Latino	UN	UN

Supplementary Table S7. Individual patient information for human cytokine analysis. Tumor type, elraglusib dose (mg, mg/kg), number of cycles, age at enrollment, sex, race, ethnicity, median progression-free survival and median overall survival data is shown (*N*=19).

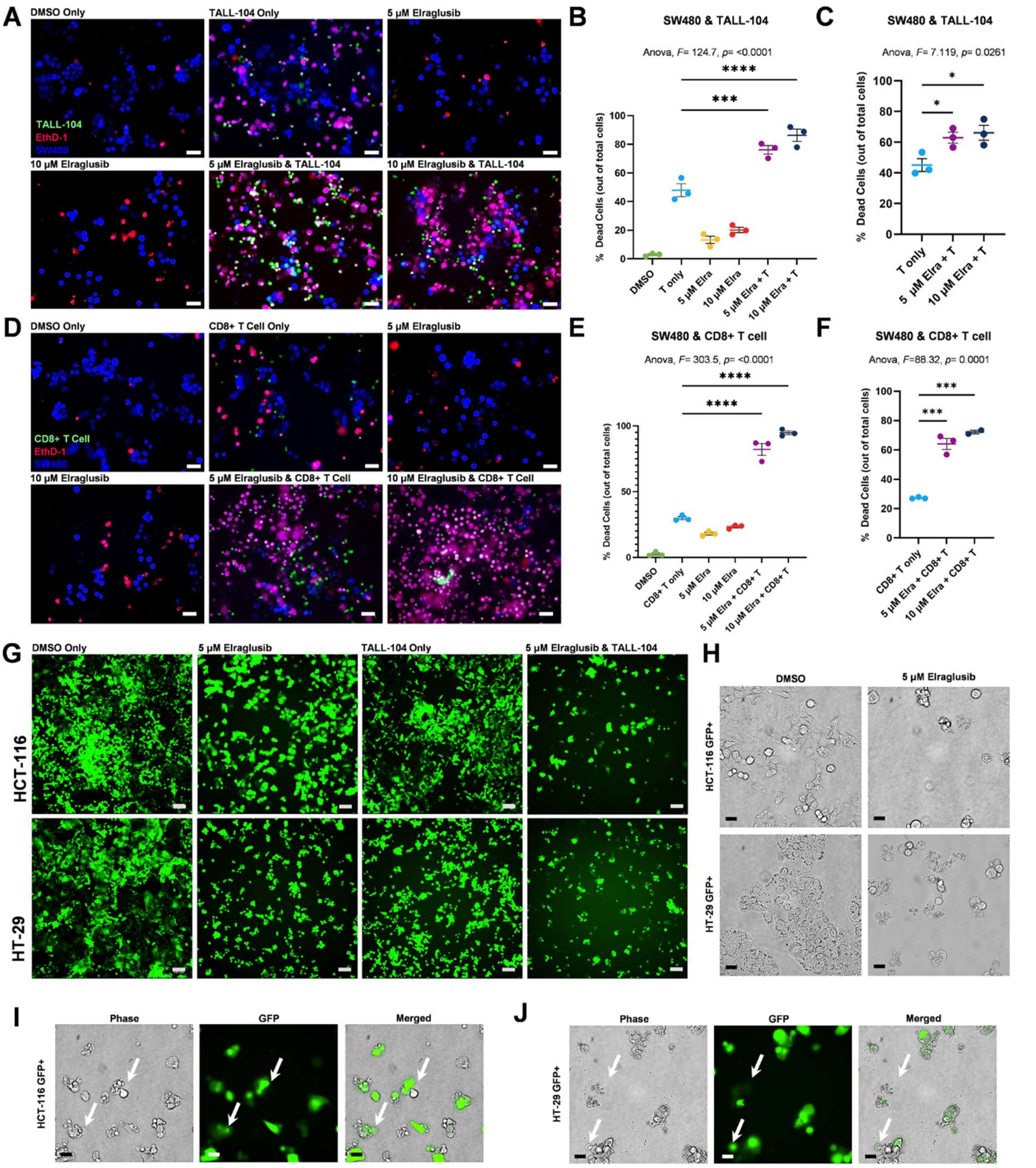

Supplementary Figure S1. Elraglusib increases immune-mediated cytotoxicity in a co-culture model with CRC cells. **(A)** Representative SW480 and TALL-104 T cell co-culture assay images at the 24-hour timepoint. 24-hour tumor cell pre-treatment with 5 µM elraglusib, followed by 24-hour co-culture. EthD-1 was used to visualize dead cells, 10× magnification, scale bar indicates 100 µm. **(B)** Quantification of co-culture experiment using the percentage of dead cells out of total cells (*N*=3). **(C)** Quantification normalized by cell death observed with drug treatment alone (*N*=3). **(D)** Representative SW480 and donor-derived CD8+ T cell co-culture assay images at the 24-hour timepoint. **(E)** Quantification of co-culture experiment using the percentage of dead cells out of total cells (*N*=3). **(F)** Quantification normalized by cell death observed with drug treatment alone (*N*=3). A one-way ANOVA followed by a post-hoc Dunnett's multiple

comparisons test was used to calculate statistical significance. **(G)** HCT-116 GFP+ or HT-29 GFP+ cells were co-cultured with TALL-104 cells at a 1:1 E:T ratio and were treated with DMSO or 5 μM elraglusib. **(H)** Representative 40× images were collected after 24 hours of DMSO or 5 μM elraglusib treatment. **(I)** Representative 40× images of a co-culture of HCT-116 GFP+ cells and NK-92 cells at a 1:1 E:T ratio were collected after 36 hours of 5 μM elraglusib treatment. White arrows indicate pyroptotic events. **(J)** Representative 40× images of a co-culture of HT-29 GFP+ CRC cells and NK-92 cells at a 1:1 E:T ratio were after 36 hours of 5 μM elraglusib treatment. White arrows indicate pyroptotic events. P-value legend: * $p < 0.05$, ** $p < 0.01$, *** $p < 0.001$, **** $p < 0.0001$.

A

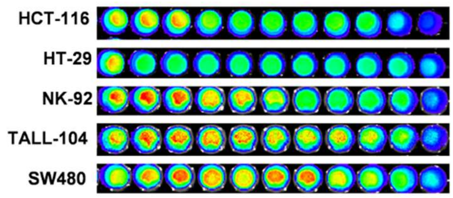

Cell Line	Elraglusib; IC_{50} (uM)
HCT-116	21.78
HT-29	100.39
NK-92	41.73
TALL-104	52.41
SW480	95.49

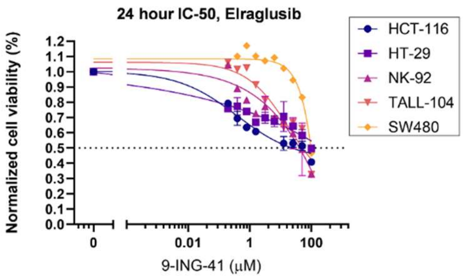

B

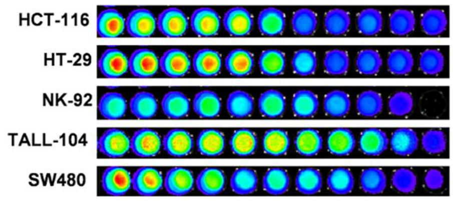

Cell Line	Elraglusib; IC_{50} (uM)
HCT-116	0.36
HT-29	0.41
NK-92	1.67
TALL-104	19.95
SW480	2.29

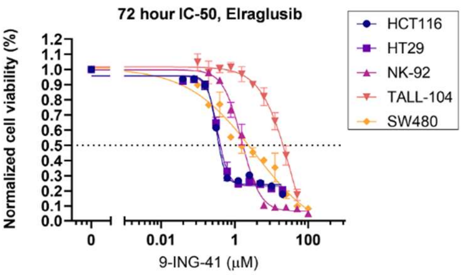

C

CRC Cell Line Name	Species	MSI / MSS status	TP53	HRAS	NRAS	KRAS	BRAF	PIK3CA	PTEN expression	APC	TRK	CTNNB1	ACVR2A	BRCA2	TGFBR2
**HCT-116**	human	MSI	WT	WT	WT	p.G13D	WT	H1047R heterozygous	positive	WT	WT	Heterozygous for p.Ser45del	p.K437fs	Heterozygous p.Ile2675fs*6	WT
**HT-29**	human	MSS	R273H	WT	WT	WT	p.V600E	P449T heterozygous	positive	E853* heterozygous, T1556fs*41	WT	WT	WT	WT	--
**KM12C**	human	MSI	p.Arg72fs*51 (c.215delG)	WT	WT	WT	WT	WT	null	Heterozygous p.Asn1819fs*7	TPM3-NTRK1 gene fusion	--	Homozygous p.Lys437fs*5	Heterozygous for BRCA2 p.Asn1784Hisfs*7	Heterozygous p.Lys128Serfs*35
**SW480**	human	MSS	R273H: P309S	WT	WT	p.G12V	WT	WT	positive	homozygous Q1338*	WT	WT	WT	WT	--

Supplementary Figure S2. CRC cell lines selected represent diverse mutational backgrounds and exhibit varying elraglusib IC-50 values. Tumor cell lines (HCT-116, HT-29, SW480) and immune cell lines (NK-92, TALL-104) were treated as indicated for **(A)** 24-hour or **(B)** 72-hour cell viability was assessed to

determine IC-50 values (*N*=3). **(C)** Table of CRC cell lines included in the study and their diverse mutational profiles.

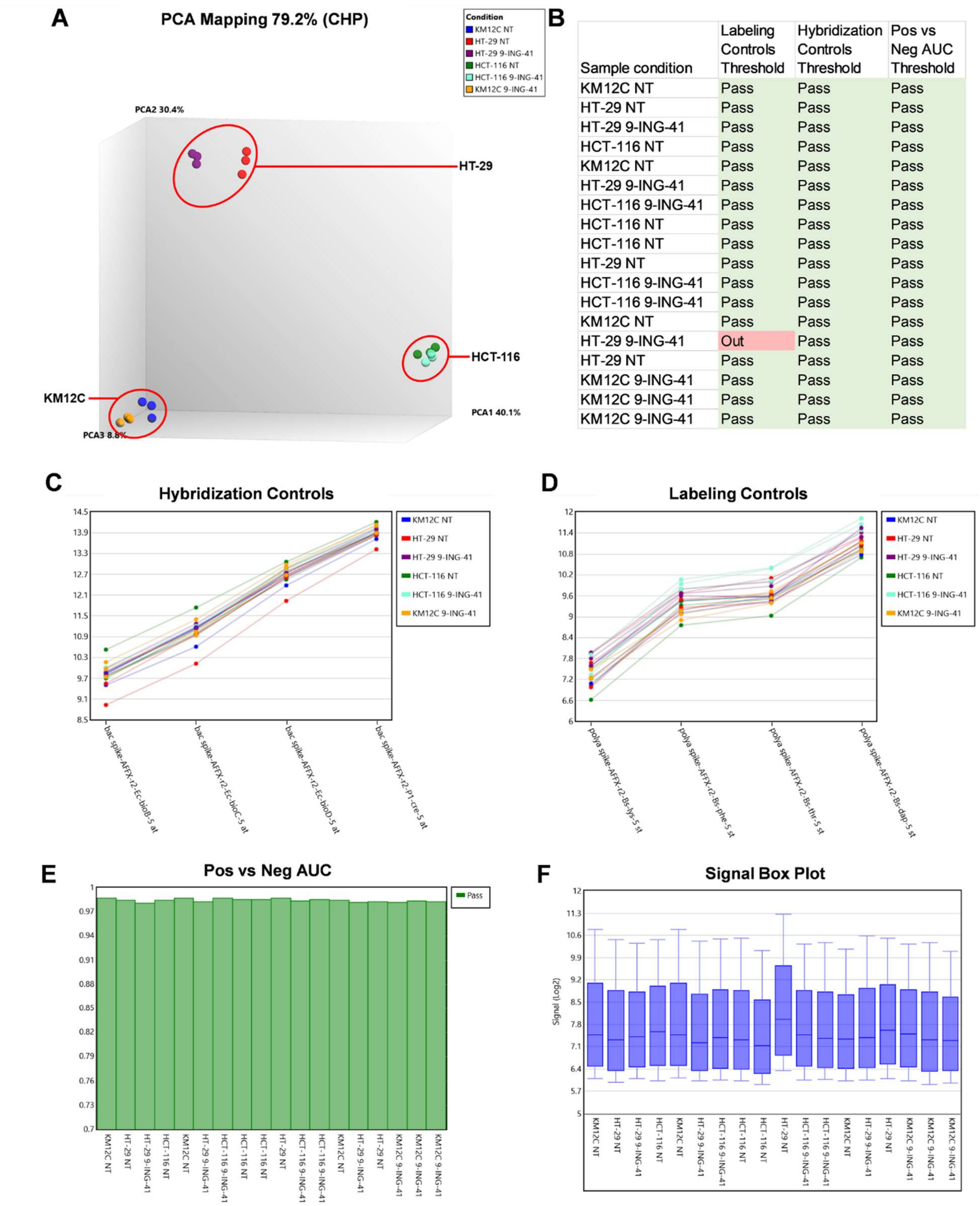

Sample condition	Labeling Controls Threshold	Hybridization Controls Threshold	Pos vs Neg AUC Threshold
KM12C NT	Pass	Pass	Pass
HT-29 NT	Pass	Pass	Pass
HT-29 9-ING-41	Pass	Pass	Pass
HCT-116 NT	Pass	Pass	Pass
KM12C NT	Pass	Pass	Pass
HT-29 9-ING-41	Pass	Pass	Pass
HCT-116 9-ING-41	Pass	Pass	Pass
HCT-116 NT	Pass	Pass	Pass
HCT-116 NT	Pass	Pass	Pass
HT-29 NT	Pass	Pass	Pass
HCT-116 9-ING-41	Pass	Pass	Pass
HCT-116 9-ING-41	Pass	Pass	Pass
KM12C NT	Pass	Pass	Pass
HT-29 9-ING-41	Out	Pass	Pass
HT-29 NT	Pass	Pass	Pass
KM12C 9-ING-41	Pass	Pass	Pass
KM12C 9-ING-41	Pass	Pass	Pass
KM12C 9-ING-41	Pass	Pass	Pass

Supplementary Figure S3. Internal controls for tumor cell microarray analysis. (A) PCA mapping demonstrated clear mapping of HCT-116, HT-29, and KM12C cells (*N*=3). **(B)** Quality control was determined satisfactory for further analysis. **(C)** Hybridization controls. **(D)** Labeling controls. **(E)** Pos vs Neg Area Under the Curve (AUC). **(F)** Signal box plot.

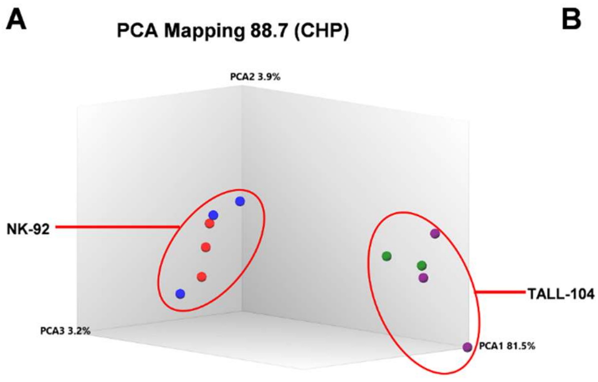

B

Sample condition	Labeling Controls Threshold	Hybridization Controls Threshold	Pos vs Neg AUC Threshold
NK-92 9-ING-41	Pass	Pass	Pass
NK-92 NT	Pass	Pass	Pass
TALL-104 9-ING-41	Pass	Pass	Pass
NK-92 NT	Pass	Pass	Pass
TALL-104 NT	Pass	Pass	Pass
NK-92 9-ING-41	Pass	Pass	Pass
NK-92 NT	Pass	Pass	Pass
TALL-104 9-ING-41	Pass	Pass	Pass
NK-92 9-ING-41	Pass	Pass	Pass
TALL-104 9-ING-41	Pass	Pass	Pass
TALL-104 NT	Pass	Pass	Pass

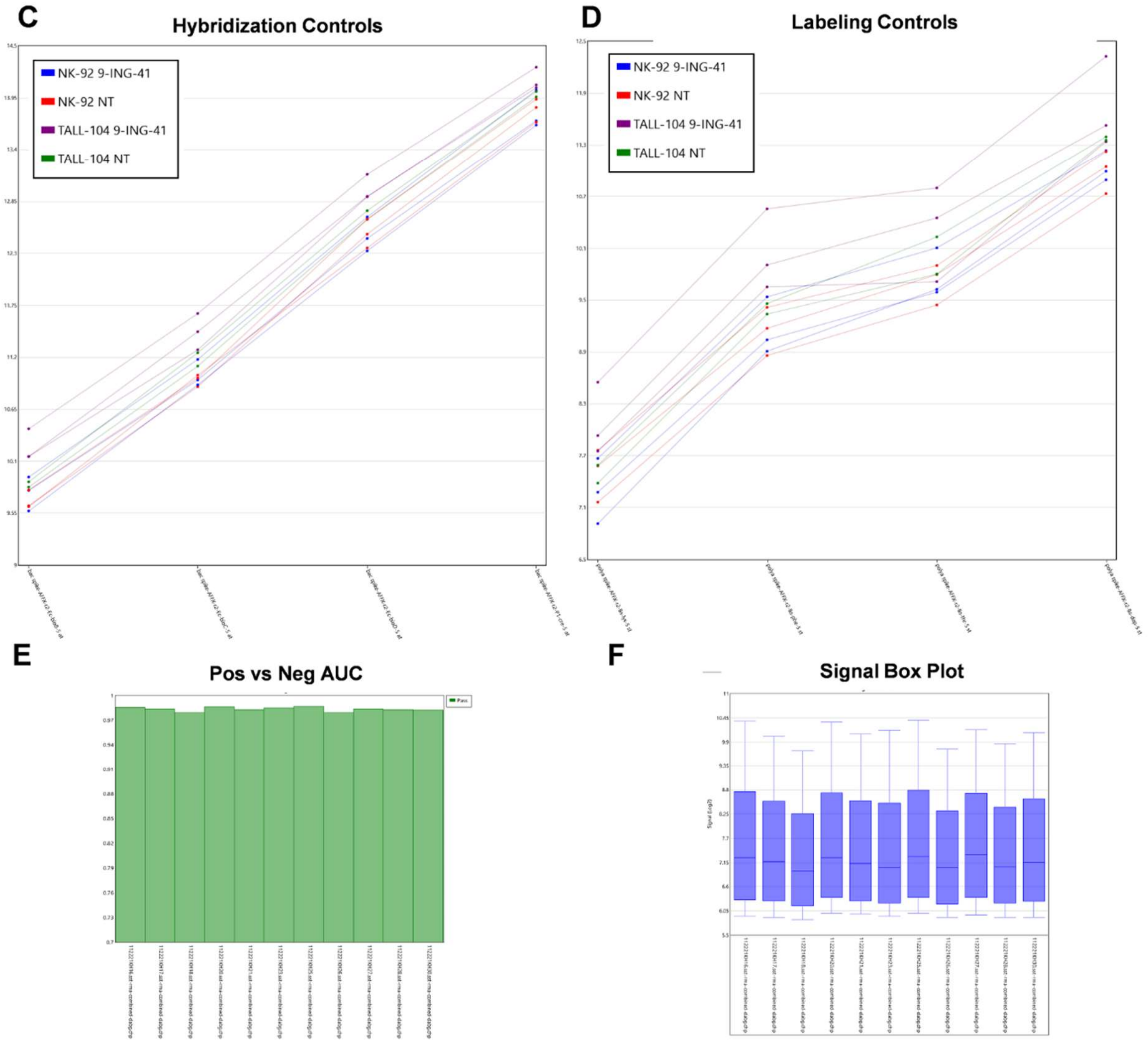

Supplementary Figure S4. Internal controls for immune cell microarray analysis. (A) PCA mapping demonstrated clear mapping of NK-91 and TALL-104 cells (*N*=3). **(B)** Quality control was determined satisfactory for further analysis. **(C)** Hybridization controls. **(D)** Labeling controls. **(E)** Pos vs Neg Area Under the Curve (AUC). **(F)** Signal box plot.

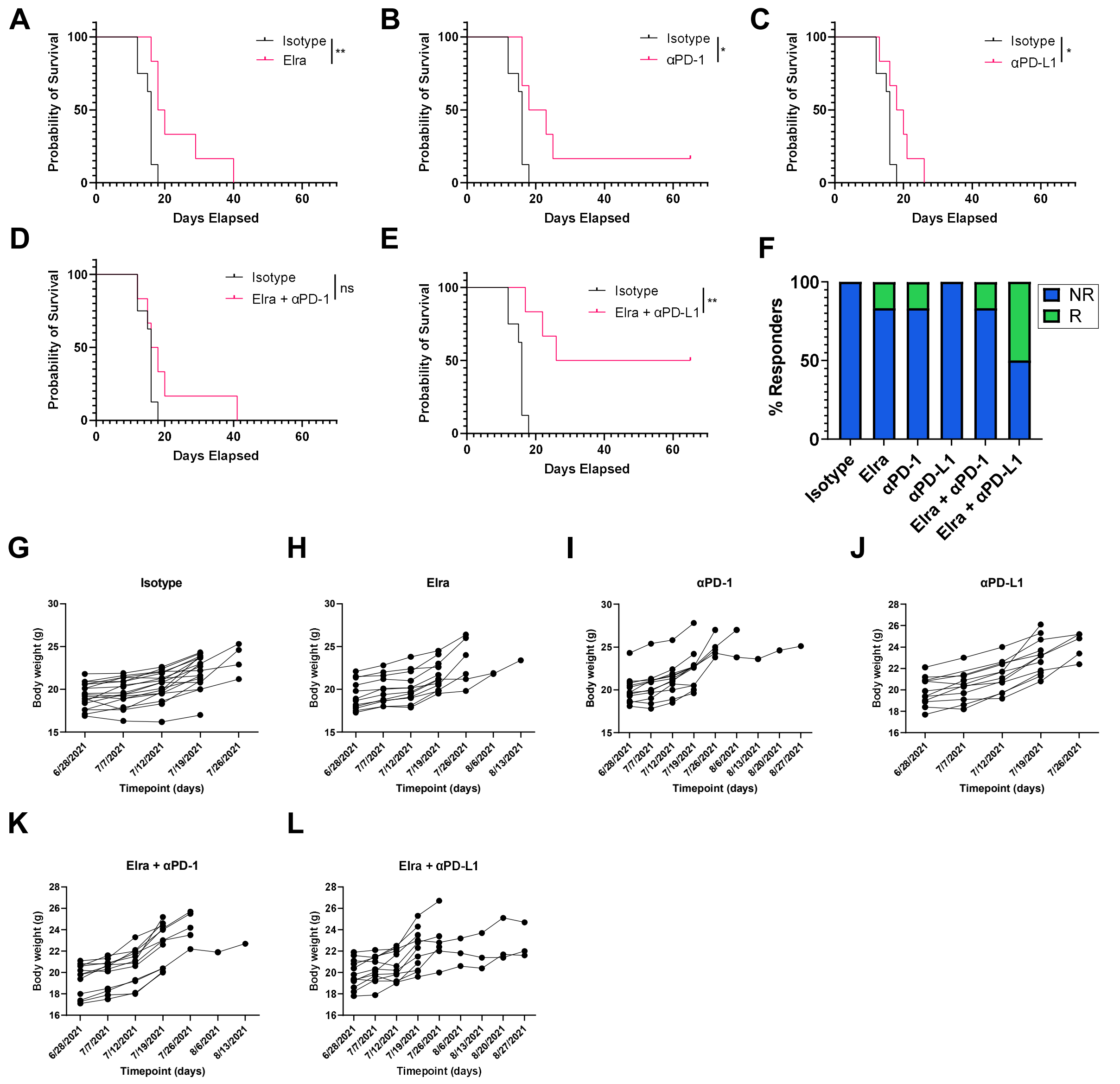

Supplementary Figure S5. Syngeneic murine colon carcinoma BALB/c murine model with MSS cell line CT-26 Kaplan Meier curves and mouse body weights grouped by treatment. Individual Kaplan Meier curves for isotype control (*N*=12) compared to **(A)** elraglusib (*N*=12), **(B)** anti-PD-1 (*N*=12), **(C)** anti-PD-L1 (*N*=12), **(D)** elraglusib + anti-PD-1 (*N*=12), and **(E)** elraglusib + anti-PD-L1 (*N*=12). **(F)** Bar graph indicating the percentage of responders (R) and non-responders (NR) per treatment group. Individual body weight plots for **(G)** Isotype control (*N*=12), **(H)** elraglusib (*N*=12), **(I)** anti-PD-1 (*N*=12), **(J)** anti-PD-L1 (*N*=12), **(K)** elraglusib + anti-PD-1 (*N*=12), and **(L)** elraglusib + anti-PD-L1 (*N*=12). P-value legend: * $p < 0.05$, ** $p < 0.01$.

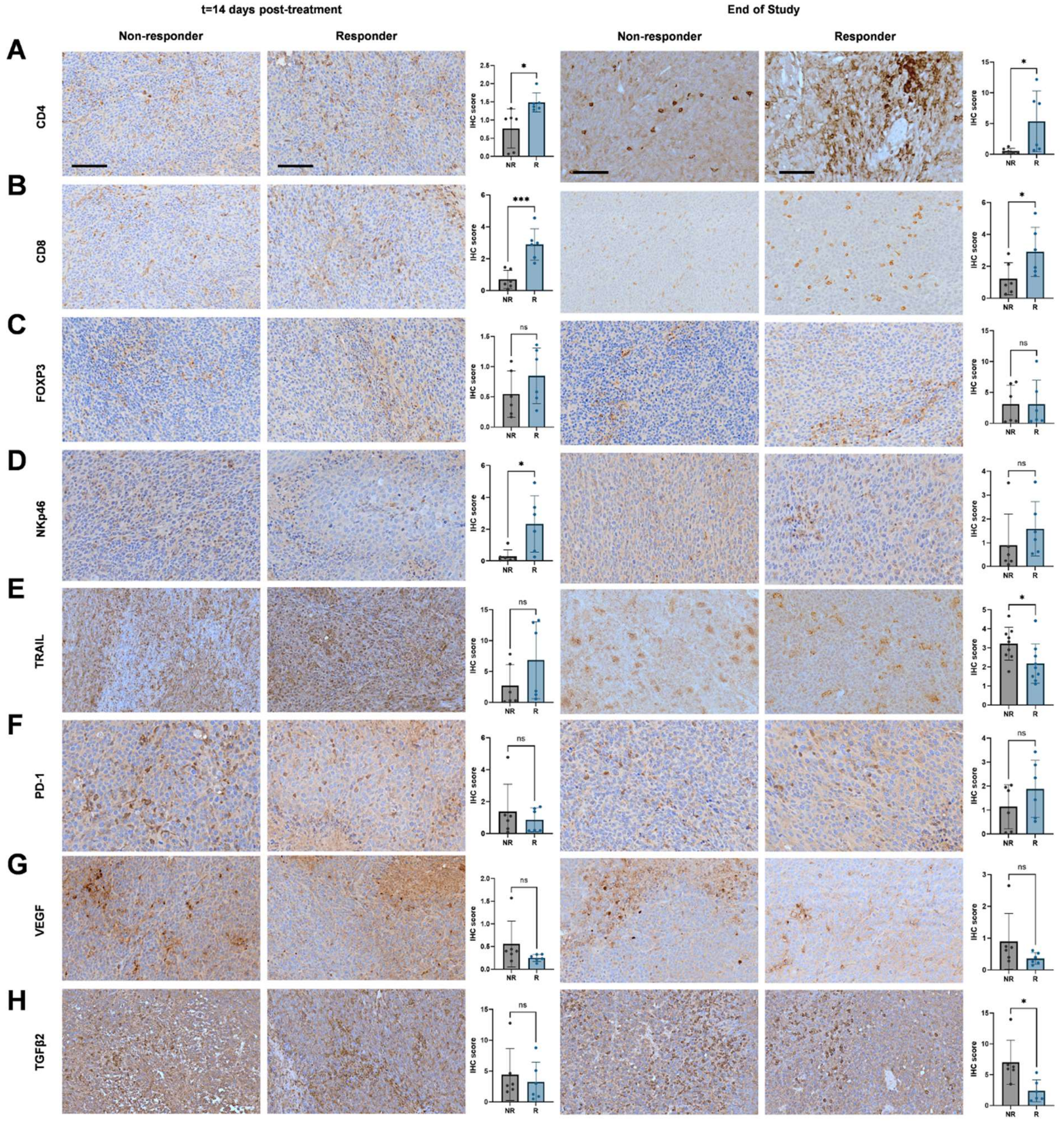

Supplementary Figure S6. Immunohistochemistry analysis of tumor sections in responders as compared to non-responders. (A) CD4, **(B)** CD8, **(C)** Foxp3, **(D)** NKp46, **(E)** TNF-related apoptosis-inducing ligand (TRAIL), **(F)** PD-1, **(G)** Vascular Endothelial Growth Factor (VEGF), and **(H)** Transforming Growth Factor Beta 2 (TGFβ2) were compared at the 14 days post-treatment initiation timepoint and the end-of-study (EOS) timepoint, respectively. Non-responders (NR, *N*=18) and responders (R, *N*=3) were compared. Statistical significance was determined using two-tailed unpaired T tests (*N*=6). 20× images, scale bar represents 100 μm.

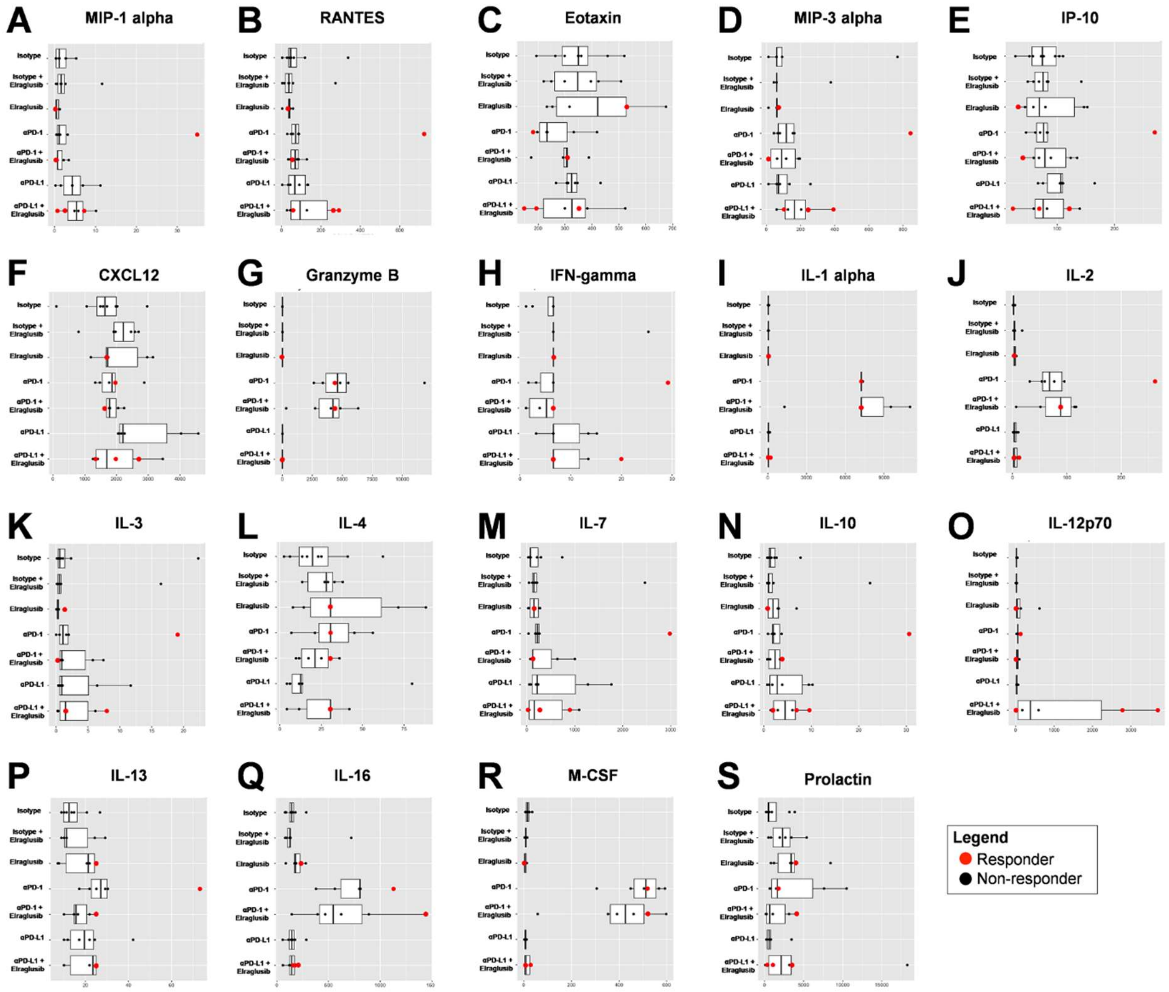

Supplementary Figure S7. Murine serum cytokine profiling assay results. Serum from end-of-study mice was analyzed via cytokine profiling for (A) MIP-1 α, (B) RANTES, (C) Eotaxin, (D) MIP-3 α, (E) IP-10, (F) CXCL12, (G) Granzyme B, (H) IFN-γ, (I) IL-1 α, (J) IL-2, (K) IL-3, (L) IL-4, (M) IL-7, (N) IL-10, (O) IL-12 p70, (P) IL-13, (Q) IL-16, (R) M-CSF, and (S) Prolactin. Responders (red, N=3) and non-responders (black, N=18) were compared. A Kruskal-Wallis test was used to calculate statistical significance followed by a Benjamini-Hochberg correction for multiple comparisons.

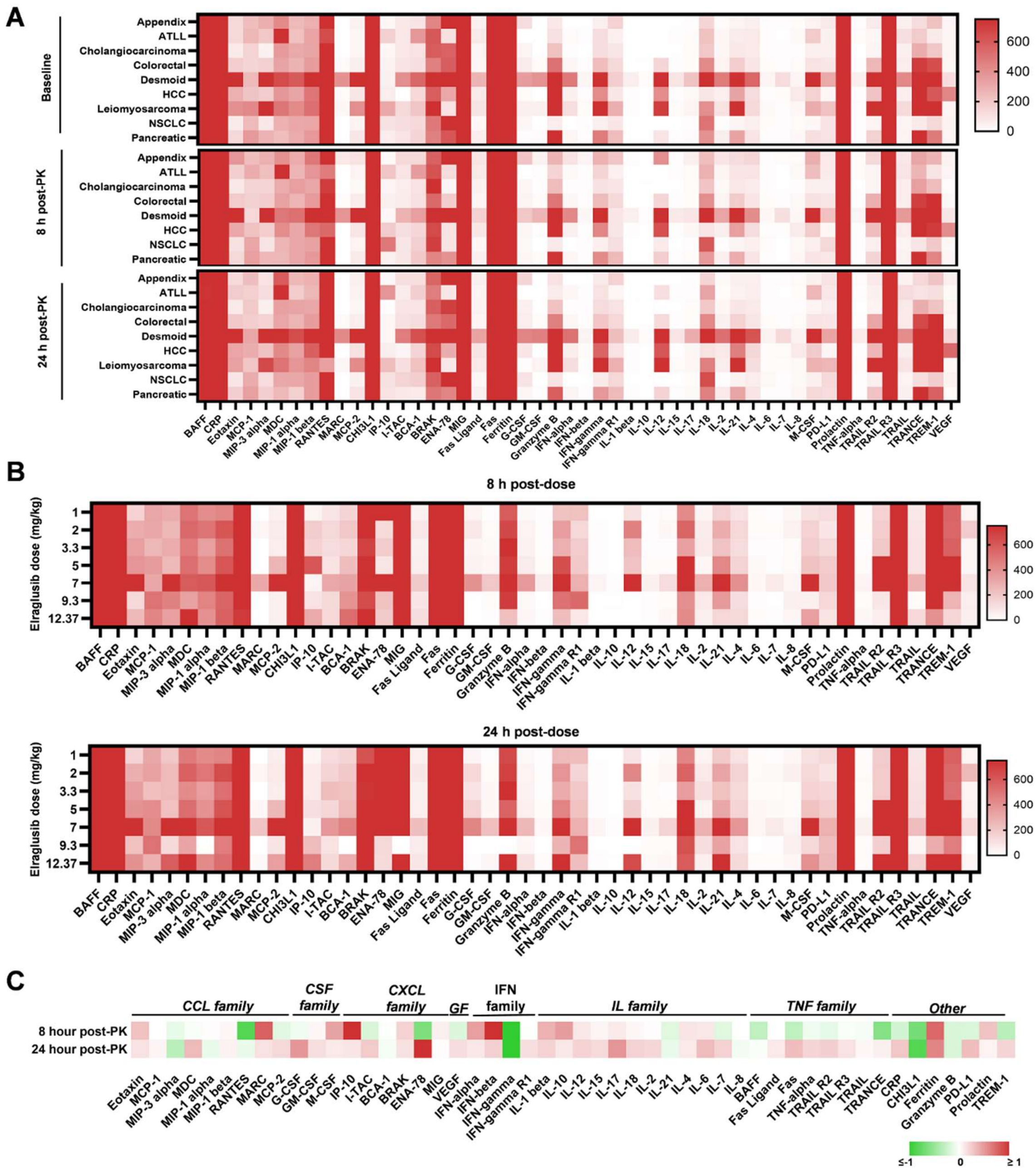

Supplementary Figure S8. Multiplex immunoassay reveals dynamic cytokine changes 8- and 24-hours post-elraglusib dose. Plasma samples from human patients with refractory solid tumors of multiple tissue origins enrolled in a Phase 1 clinical trial investigating GSK-3 inhibitor elraglusib (NCT03678883) were analyzed using a Luminex 200 (*N*=19). **(A)** Raw analyte values are grouped by timepoint: baseline (pre-dose), 8-hour post-PK, and 24-hour post-PK and categorized by primary tumor location: appendix, adult T cell leukemia/lymphoma (ATLL), cholangiocarcinoma, colorectal, desmoid, hepatocellular carcinoma (HCC), leiomyosarcoma, non-small cell lung cancer (NSCLC), and pancreatic. The scale bar is in pg/mL. **(B)** Raw analyte values are grouped by timepoint: 8-hour post-PK and 24-hour post-PK and categorized by elraglusib dose: 1, 2, 3.3, 5, 7, 9.3, or 12.37 mg/kg. **(C)** Fold change heatmaps grouped by cytokine family.

8- and 24-hours post-PK were compared to baseline (pre-PK) analyte values. Green indicates downregulation and red indicates upregulation.

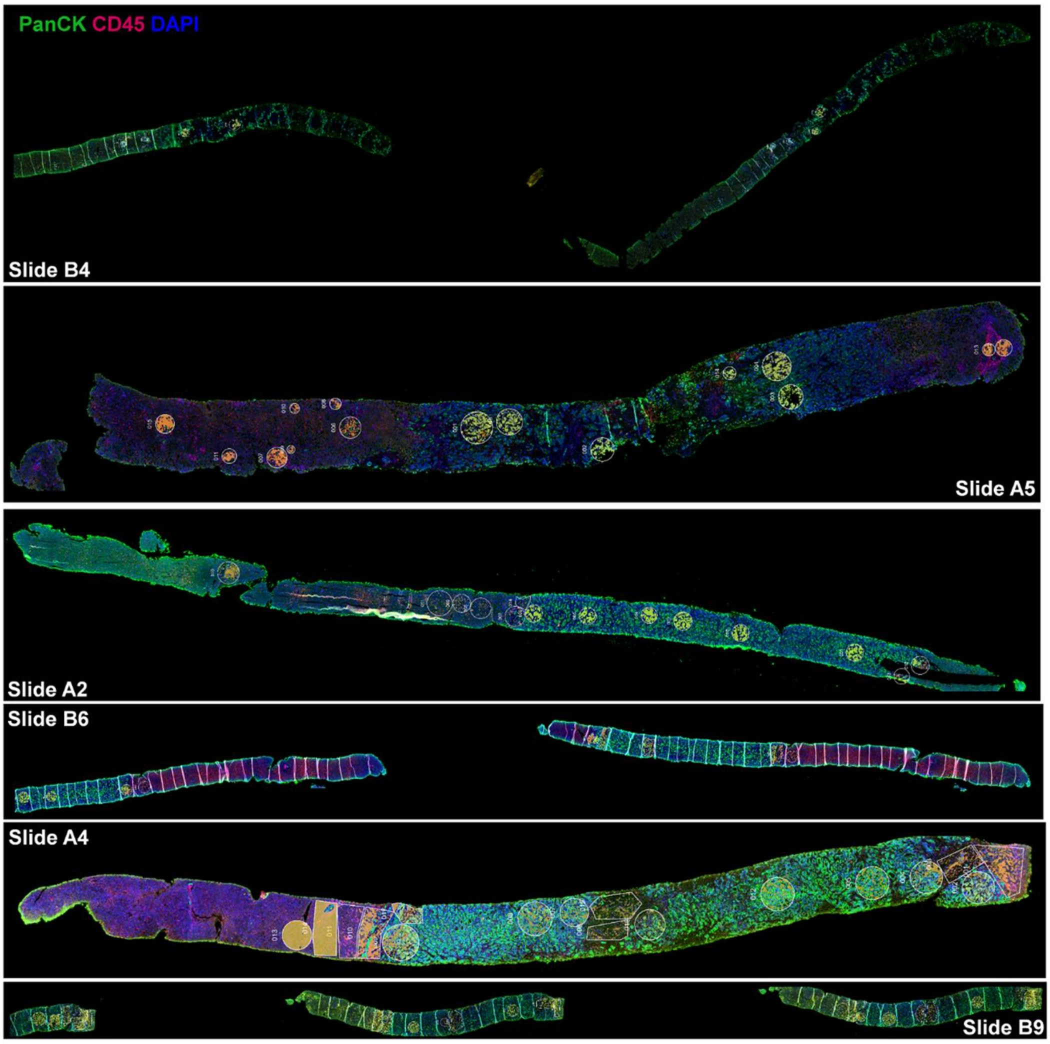

Supplementary Figure S9. Needle biopsies scanned by the GeoMx Digital Spatial Profiler (DSP). Whole slide scans are provided for all needle biopsies imaged by the GeoMx DSP (*N*=6 biopsies). PanCK is indicated by green, CD45 is indicated by red, and DAPI is indicated by blue staining. Slide IDs are provided on each image in white text.

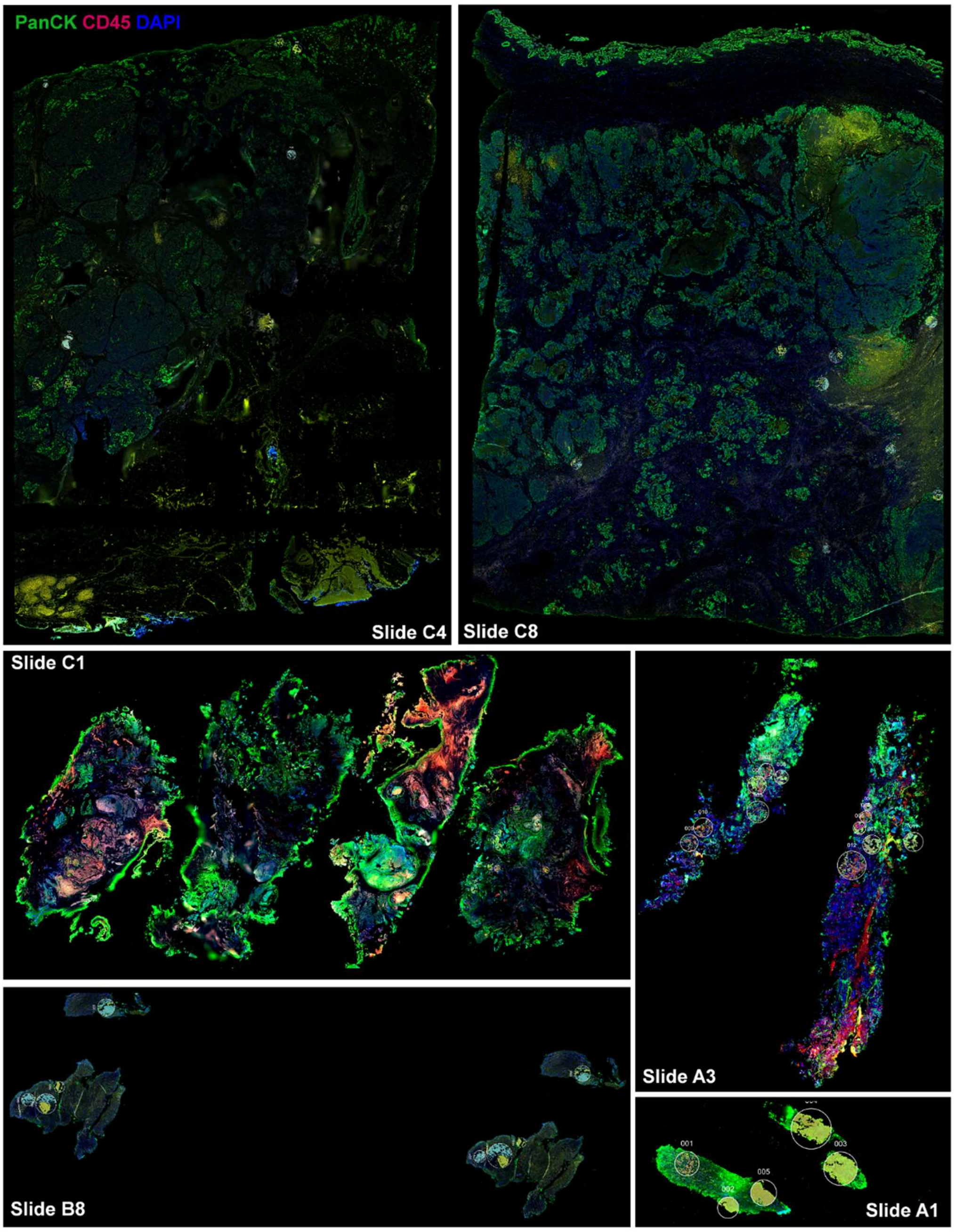

Supplementary Figure S10. Tissue biopsies scanned by the GeoMx Digital Spatial Profiler (DSP). Whole slide scans are provided for all tissue biopsies imaged by the GeoMx DSP (*N*=6 biopsies). PanCK is indicated by green, CD45 is indicated by red, and DAPI is indicated by blue staining. Slide IDs are provided on each image in white text.

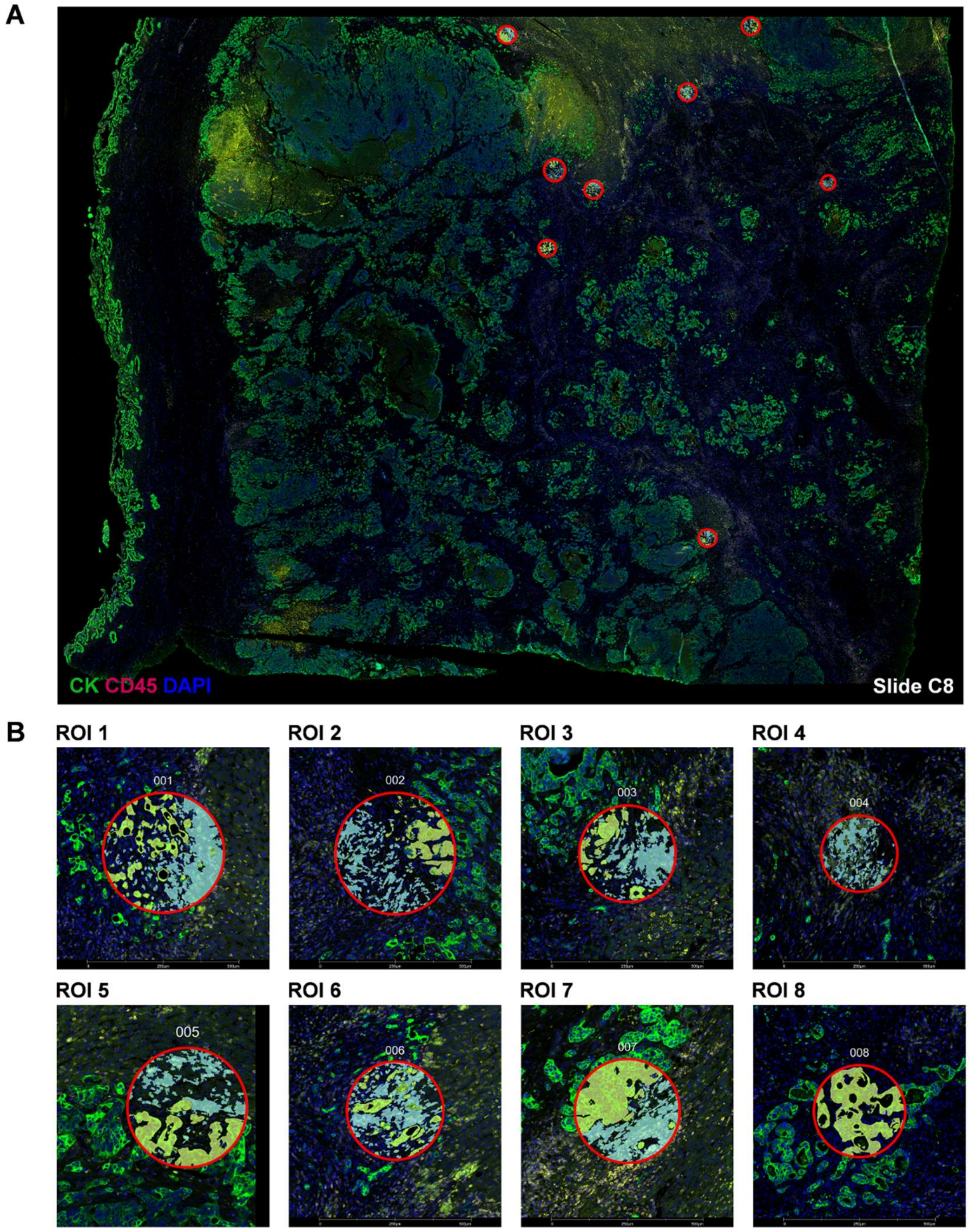

Supplementary Figure S11. ROI selection and segmentation strategy using PanCK and CD45 markers. **(A)** Representative image of whole tissue scan used for ROI identification. ROIs are outlined in red circles. **(B)** Individual ROIs are ordered by number. Fluorescent channel settings: FITC / 525nm / SYTO 13 / DNA (Blue), Cy3 / 568nm / Alexa 532 / PanCK (Green), and Texas Red / 615nm / Alexa 594 / CD45 (Red). A yellow mask was used for PanCK+ tumor cell identification and a teal mask was used for CD45+ hematopoietic cell identification.

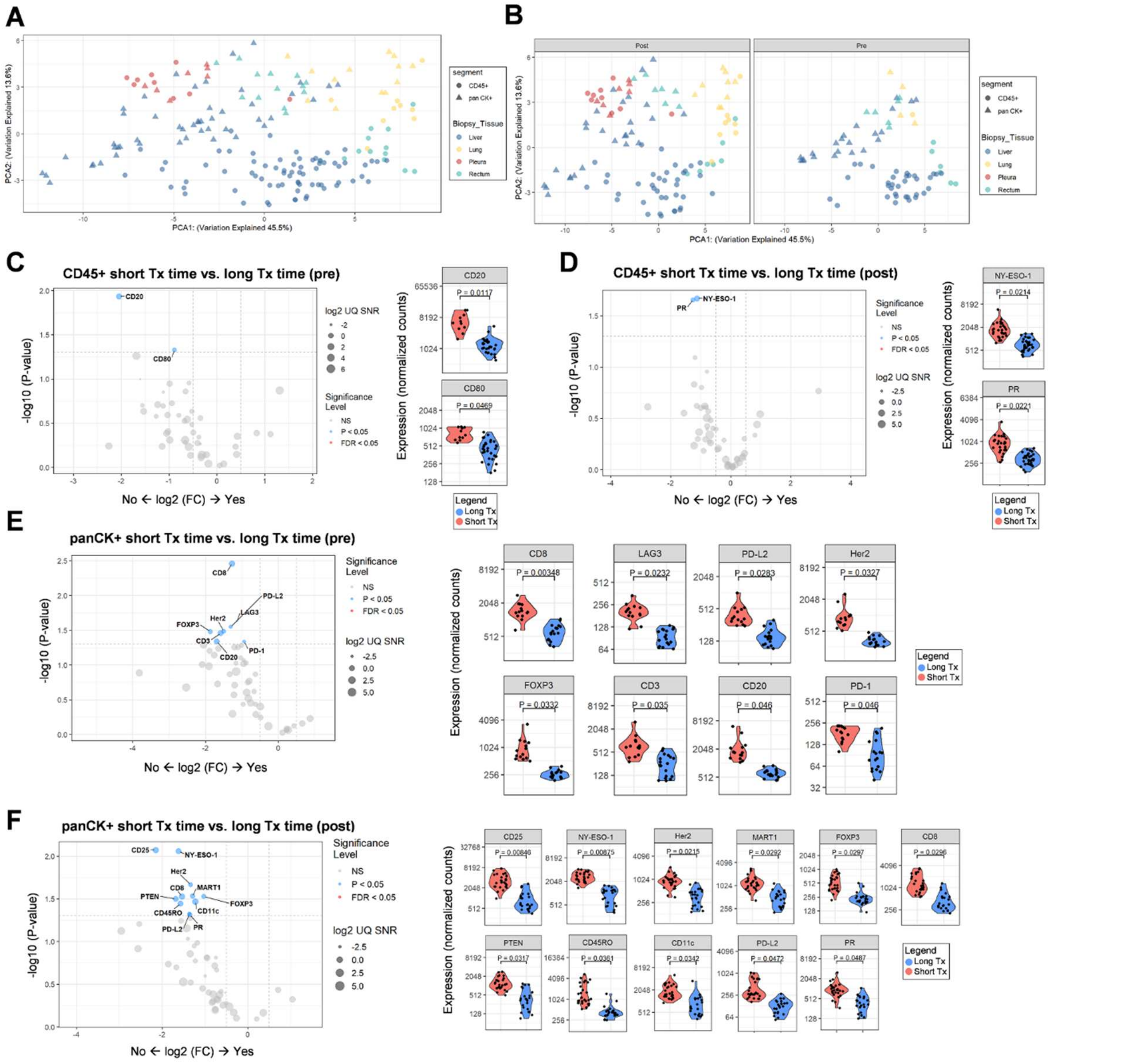

Supplementary Figure S12. Several proteins are differentially expressed when patients are segmented into two groups based on time-on-treatment. PCA plots showing how similar the different group levels are from one another in the **(A)** total sample set (*N*=12) and **(B)** at both biopsies timepoints [pre- (*N*=5), post-treatment, (*N*=7). Samples tend to cluster by tissue type and further separate by segment on PC2. Circles represent CD45+ segments and triangles represent pan CK+ segments. Biopsy tissue locations are color-coded where blue indicates liver, yellow indicates lung, red indicates pleura, and green indicates rectum. **(C)** Volcano plot showing the comparison of pre-treatment biopsy protein expression in CD45+ segments between long time-on-treatment (Long Tx) patients and short time-on-treatment (Short Tx) patients. Violin plots show statistically significant differentially expressed proteins including B cell marker CD20 (*p*=0.012) and myeloid activation marker CD80 (*p*=0.047). **(D)** Volcano plot showing the comparison of CD45+ segments in post-treatment biopsies between Long Tx patients and Short Tx patients. Violin plots show statistically significant differentially expressed proteins including antigen NY-ESO-1 (*p*=0.021) and progesterone receptor (PR) (*p*=0.022). **(E)** Volcano plot showing the comparison of pre-treatment protein expression in PanCK+ segments between Long Tx patients and Short Tx patients. Violin plots show

statistically significant differentially expressed proteins including cytotoxic T cell marker CD8 (p=3.5E-3), antigen Her2 (p=0.033), Treg marker Foxp3 (p=0.033), T cell marker CD3 (p=0.035), B cell marker CD20 (p=0.046), LAG3 (p=0.023), PD-L2 (p=0.028), and PD-1 (p=0.046). **(F)** Volcano plot showing the comparison of post-treatment protein expression in PanCK+ segments between Long Tx patients and Short Tx patients. Violin plots show statistically significant differentially expressed proteins including mature B cell/DC marker CD35 (p=8.5E-3), antigen NY-ESO-1 (p=8.7E-3), antigen Her2 (p=0.022), antigen MART1 (p=0.029), cytotoxic T cell marker CD8 (p=0.030), Treg marker Foxp3 (p=0.030), antigen PTEN (p=0.032), DC/myeloid marker CD11c (p=0.034), memory T cell marker CD45RO (p=0.036), checkpoint PD-L1 (p=0.047), and PR (p=0.049).

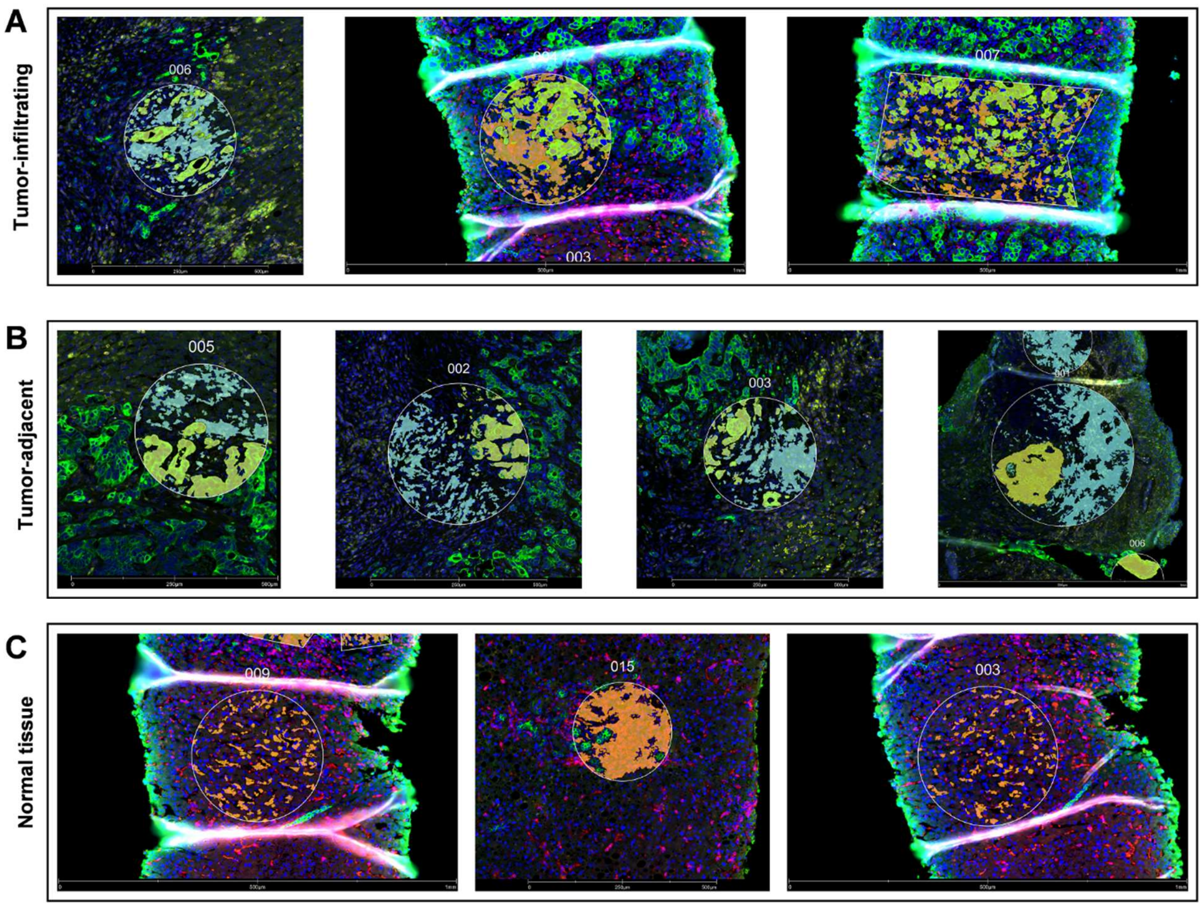

Supplementary Figure S13. Immune cell localization categorization strategy. Immune cell locations were categorized as **(A)** tumor-infiltrating, **(B)** tumor-adjacent, or **(C)** normal tissue. Representative ROI images are shown.

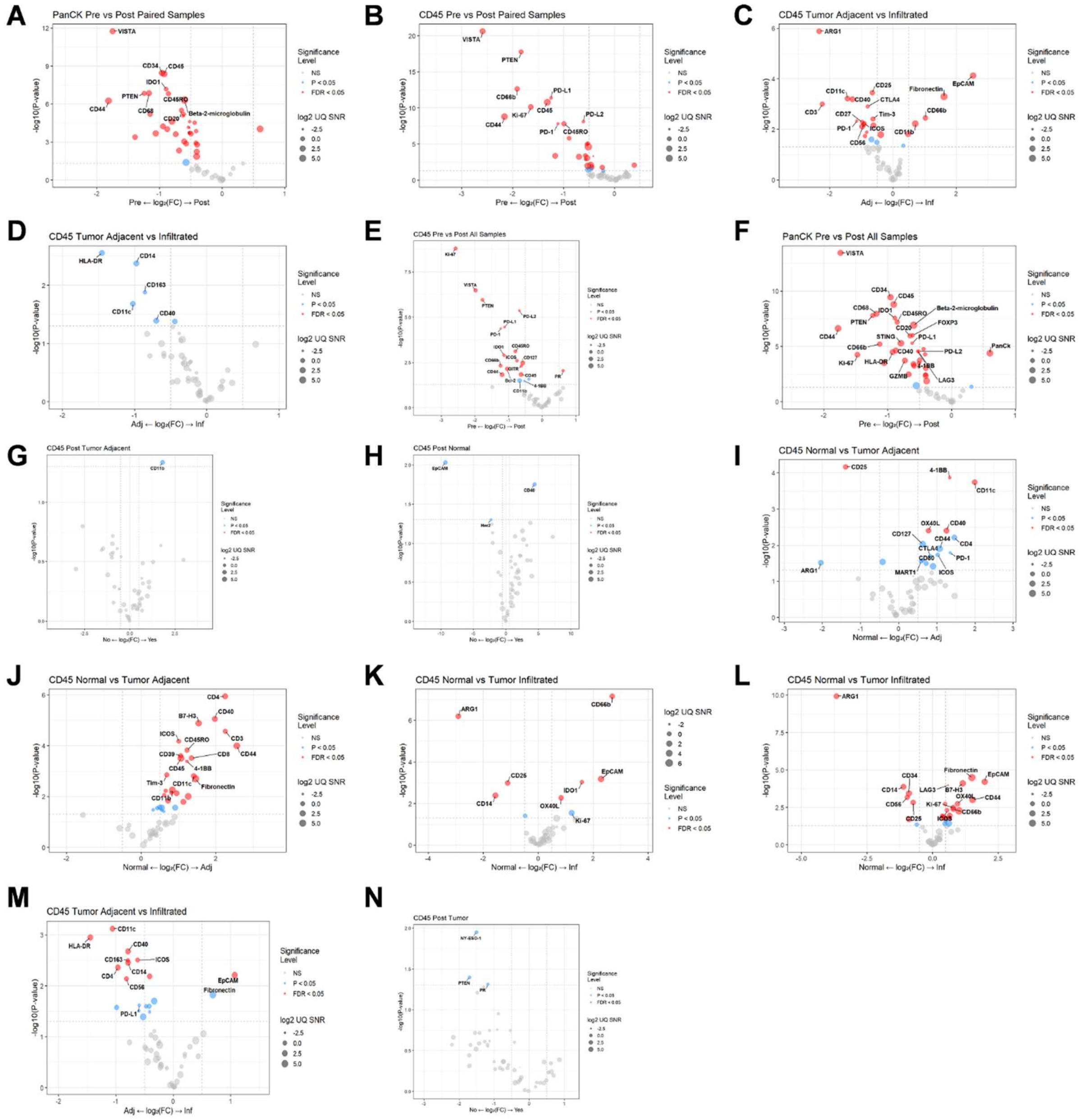

Supplementary Figure S14. Differential expression analyses of protein expression in human tumor biopsies pre- and post-elraglusib treatment. (A) Volcano plot showing differential protein expression in PanCK+ regions between pre- (*N*=5) and post-treatment (*N*=7) biopsies in paired samples. **(B)** Volcano plot showing differential protein expression in CD45+ regions between pre- and post-treatment biopsies in paired samples. **(C)** Volcano plot showing differential protein expression between tumor-adjacent CD45+ segments and tumor-infiltrating CD45+ segments in pre-treatment biopsies. **(D)** Volcano plot showing differential protein expression between tumor-adjacent CD45+ segments and tumor-infiltrating CD45+ segments in post-treatment biopsies. **(E)** Volcano plot showing differential protein expression between CD45+ regions of pre-treatment and post-treatment biopsies. **(F)** Volcano plot showing differential protein expression panCK+ regions of pre-treatment and post-treatment biopsies. **(G)** Volcano plot showing differential post-treatment protein expression in tumor-adjacent CD45+ immune cell segments in Long Tx patients as compared to Short Tx patients. **(H)** Volcano plot showing differential post-treatment protein

expression in CD45+ immune cell segments located in normal (non-tumor) tissue in Long Tx patients as compared to Short Tx patients. **(I)** Volcano plot showing differential pre-treatment protein expression in CD45+ immune cell segments located in normal (non-tumor) tissue as compared to tumor-adjacent tissue. **(J)** Volcano plot showing differential post-treatment protein expression in CD45+ immune cell segments located in normal (non-tumor) tissue as compared to tumor-adjacent tissue. **(K)** Volcano plot showing differential pre-treatment protein expression in CD45+ immune cell segments located in normal (non-tumor) tissue as compared to those in tumor tissue. **(L)** Volcano plot showing differential post-treatment protein expression in CD45+ immune cell segments located in normal (non-tumor) tissue as compared to those in tumor tissue. **(M)** Volcano plot showing a comparison of tumor-adjacent CD45+ segments with tumor-infiltrating CD45+ segments in all biopsies regardless of timepoint. **(N)** Volcano plot showing a comparison of post-treatment protein expression of tumor-infiltrating CD45+ immune cell segments in Long Tx patients as compared to Short Tx patients. Grey points are non-significant (NS), blue points have p values < 0.05, and red points have false discovery rate (FDR) values less than 0.05. The size of the point represents the log2 UQ Signal-to-noise ratio (SNR).
